# Magic Ring: A Finger-Worn Device for Multiple Appliances Control Using Static Finger Gestures

**DOI:** 10.3390/s120505775

**Published:** 2012-05-04

**Authors:** Lei Jing, Yinghui Zhou, Zixue Cheng, Tongjun Huang

**Affiliations:** 1 School of Computer Science and Engineering, University of Aizu, Tsuruga, Ikki-machi, Aizu-Wakamatsu City, Fukushima, 965-8580, Japan; E-Mails: z-cheng@u-aizu.ac.jp (Z.C.); t-huang@u-aizu.ac.jp (T.H.); 2 Graduate School of Computer Science and Engineering, University of Aizu, Tsuruga, Ikki-machi, Aizu-Wakamatsu City, Fukushima, 965-8580, Japan; E-Mail: d8131104@u-aizu.ac.jp

**Keywords:** internet of things, human-centric sensing, gestural interface, remote control, wearable, gesture recognition

## Abstract

An ultimate goal for Ubiquitous Computing is to enable people to interact with the surrounding electrical devices using their habitual body gestures as they communicate with each other. The feasibility of such an idea is demonstrated through a wearable gestural device named Magic Ring (MR), which is an original compact wireless sensing mote in a ring shape that can recognize various finger gestures. A scenario of wireless multiple appliances control is selected as a case study to evaluate the usability of such a gestural interface. Experiments comparing the MR and a Remote Controller (RC) were performed to evaluate the usability. From the results, only with 10 minutes practice, the proposed paradigm of gestural-based control can achieve a performance of completing about six tasks per minute, which is in the same level of the RC-based method.

## 1. Introduction

In the vision of cyber-physical world or Internet of Things (IoT), networked digital artifacts, like smart buildings, smart desks, smart bags, and so on, will be densely deployed in people's everyday life [1]. It is impractical to control all of these artifacts through physical touch on buttons or touchscreens. As an alternative way, wearable gestural interfaces allow humans to interact with these artifacts by natural body gestures. This removes the above extra equipment. Moreover, if an effective gesture vocabulary set was defined, people could interact with different kinds of digital artifacts in a unified way. Thus they could become familiar with a new ubiquitous environment with lower learning efforts and interact with surrounding digital artifacts with lower cognitive load.

Researchers have shown the feasibility of detecting the diverse activities of almost all of the body parts, including foot [2–4], shoulder [5], arm [6–8], hand [8–11] by using various body-worn sensors. These researches confirmed that a reasonable recognition rate can be achieved in either online or off-line processing [12,13].

However, little research has been performed on detection of the gestures of a single finger using wearable sensors. Obviously, sensors put on one finger are more lightweight and can detect the subtle gestures of the finger, which is difficult for other wearable devices. Meanwhile, it can detect most of the movements of the hand/wrist/arm as well.

Unobtrusiveness for other activities and usability are the two main challenges for the design of wearable sensing motes. Compared with limbs, fingers are more flexible and more frequently used in daily life. Thus, it is a challenge to effectively detect the finger gestures and avoid obstructing other jobs. Affordbility is another challenge which exists in all types of wearable devices. Wear and play is not feasible yet for most of the proposed wearable solutions since the recognition rate is sensitive to many factors like specific position and orientation of the body-worn sensors. Taking the digital glove as an example, it is a kind of well-known tools for virtual reality research. But people are reluctant to consistently wear it since it will hinder the usage of hands. Moreover, people have to adjust the digital glove to fit their hand before each time they want to use it.

From the above analysis, great progress has ocurred in wearable gesture detection field, but more effort is still needed to apply these technologies into everyday life.

In this paper, a wearable device named Magic Ring (MR) is presented for multiple appliance control. MR is a ring-type sensing mote which can be worn onto the finger to detect the finger action by sensors, recognize the finger gestures, encode gestures into control commands, and transport the commands to interactive targets. Moreover, a proof of concept system for wireless multiple appliance control is designed and implemented to demonstrate the feasibility of such an interactive paradigm. Finally, the usability of the MR is studied through comparison experiments between the MR and a traditional Remote Controller (RC).

## 2. Related Work

Speech and body language are two habitual means for human communication. Speech is a natural and convenient interface. So far, speech recognition can achieve more than 98% accuracy in participant-dependent training and testing. But in an environment with noise such as TV in the room, it is hard to recognize the user's voice separate from the background sound. Moreover, some seniors cannot pronounce clearly due to the degradation of speech organs.

Gestures are expressive body language including physical movements of some parts of body. Some previous research has shown that gestures are natural for humans, and only a short time of training is required before people can consistently use new gestures to communicate information or control devices [14–16].

Gestural interfaces can be divided into two categories, according to the sensing technology used to detect the gesture. One category adopts non-contact sensors like Charge Coupled Device (CCD) cameras [17], ultrasonic [18], and infrared sensors [19] and combined with vision recognition methods. Another one adopts contact sensors like accelerometer [20], gyroscope [21], magnetic sensors [22], and non-invasive electroencephalograms (EEGs) sensors, which has to be worn onto the body to work.

The non-contact gestural interface is lightweight for the users since people do not need to wear additional devices or some marks on the body are enough [11], but it is onerous for the system since the computational complexity for signal processing and gesture recognition is high [23]. Meanwhile, high computing costs also lead to difficulty in meeting some Quality of Service (QoS) requirements like real-time control. Recently, some great self-contained portable vision-based gestural interface is proposed like sixth sense [9]. But so far, it can only detect the two dimension gestures. It still faces the computing complexity and real-time problem if they try to detect the highly dynamic or three dimension gestures.

Unlike vision, the wearable-sensor-based method requires relatively small computing resources, even for the dynamic gestures [20]. Moreover, it is possible to detect three-dimensional natural gestures in daily life through the combination and deployment of well-designed sensors [12,13].

After a comparison between several kinds of technologies, including infrared and wireless, voice and gesture, vision and wearable, the wireless wearable sensing method was adopted for the pilot implementation of the wireless appliance control system. Wearable controllers do not need to be held in the hand, and can provide at-hand service with short accessing times. Moreover, wireless can provide two-way communication among multiple devices at the same time.

## 3. Conceptual Architecture

**Figure 1.**
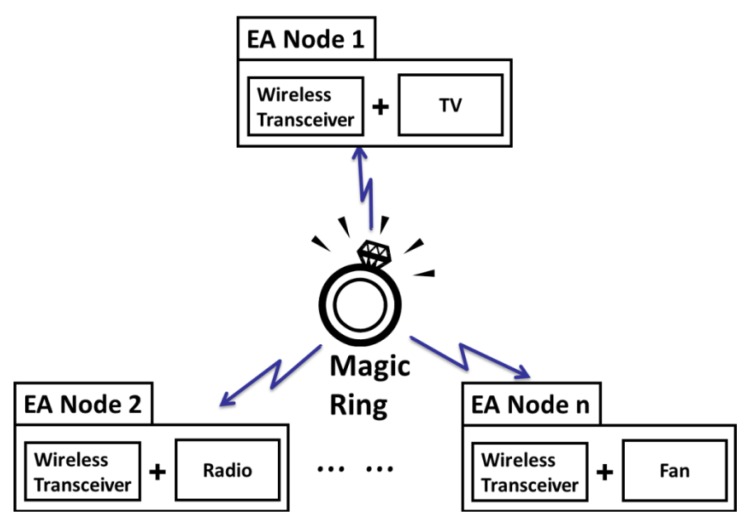
Model of finger gesture-based one-to-many remote control.

The indoor network for appliances control is a typical Wireless Personal Area Network (WPAN). Multiple appliances together with the remote controller like Magic Ring (MR) form a star topology as shown in Figure 1.

The controller (MR) is at the central node acting as the master. The appliances (like TV, Radio, Fan, and so on) are at the peripherals acting as slaves. For one-to-many control, various control targets are abstracted as one type of device called Electrical Appliance Node (EA-Node). EA-Node, as one of the two primary elements of the interface, is a traditional electrical appliance augmented with a Receiver, a component to decode and execute the control commands. Another primary element is a wearable controller, like the MR, a controller worn on a finger to identify, encode, and send the control commands.

**Figure 2.**
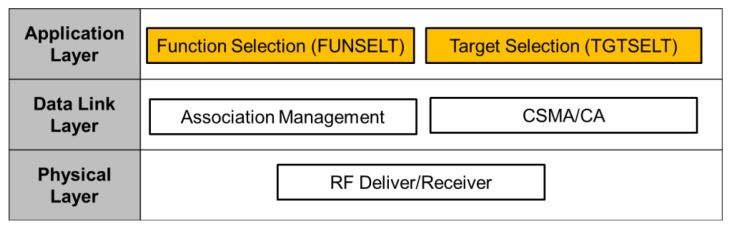
Three-layer wireless control interface.

Furthermore, a unified wireless control interface between MR and EA-Nodes is defined to enable the transparent control across the functional diversity of EA-Nodes. A three-layer software interface is defined as shown in Figure 2. The mapping relationship between the three-layer interface and standard OSI network architecture is shown as well. The bottom layer is a physical layer which is responsible for package transfer and reception through RF waves. The middle Link Layer consists of two components: Association Management and Carrier Sense Multiple Access/Collision Avoidance (CSMA/CA). The top is the application layer, which include target selection control (TGTSELT) and functional selection control (FUNSELT). TGTSELT defines the switching protocol between multiple appliances. FUNSELT is responsible for command encoding and decoding for the selection of specific functions on a selected appliance. Network and transport layer are not included into the interface since they are not necessary needed in a single hop network.

In the wireless control process, protocols of physical layer and data link layer can adopt other existing protocols of WPAN like 804.15.4 and Bluetooth. Thus, the bottom and middle layer will not be discussed in this paper, but the two special functions in the application layer, including target selection and function selection, will be discussed in more detail in Section 6.

## 4. Structure of the MR and EA-Node

### 4.1. Overall Structure Design

**Figure 3.**
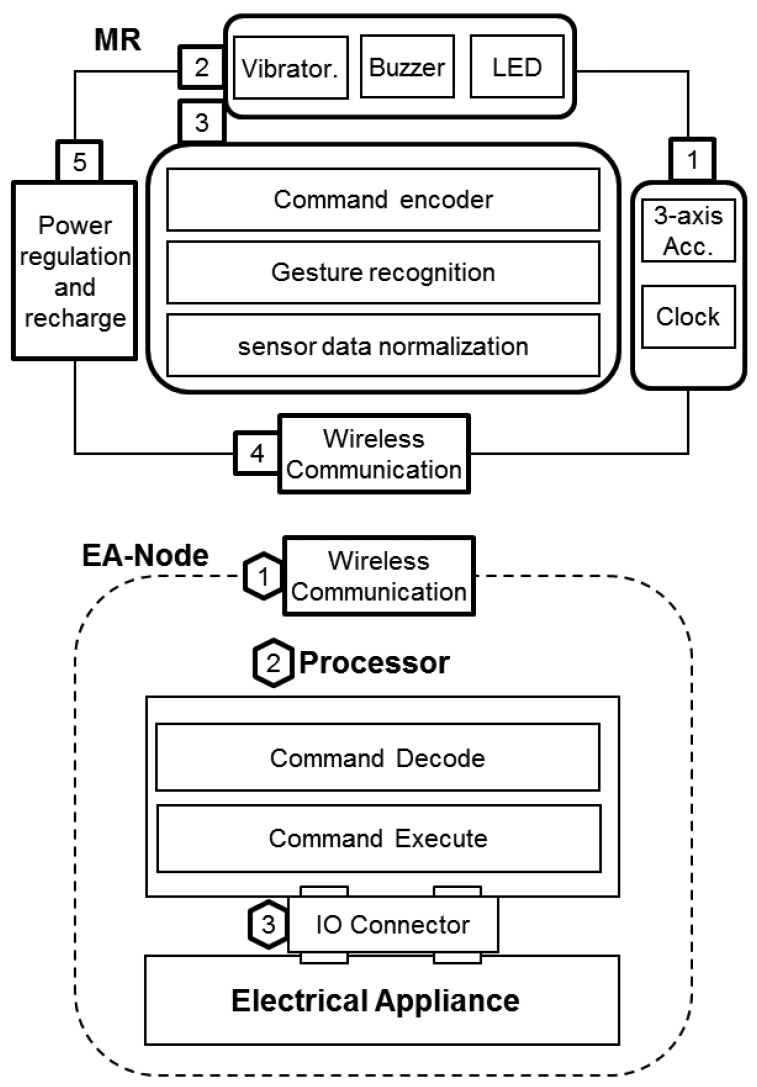
Structure of Magic Ring (MR) and EA-Node: two elements of finger-worn gestural interface.

The overall architecture consists of two kinds of main components: the MR and EA-Node, as shown in Figure 3 MR is a finger-gesture-based controller to identify, encode and send the control commands. EA-Node is a normal electrical appliance augmented with a Receiver, an interface to decode and execute the control commands. A star network topology is adopted for one-to-many control since it is simple to add or remove a node. MR is the central node, and as the control targets, EA-Nodes are the peripheral nodes. Below, a specific implementation of MR and EA-Node are introduced in more details.

### 4.2. Hardware Structure of MR

MR is used to recognize finger gestures and send control command. As shown in the upper part of Figure 3, the MR consists of five major modules: (1) sensor module (input); (2) multimodal feedback module (output); (3) processing module; (4) wireless transceiver module; and (5) power regulation and charging module.

A brief introduction of the specific implementation for the experimental system is given as follows. Sensor module including a three axis micromachined analog accelerometer MMA7361L and Real Time Clock (RTC); Multimodal feedback module (Vibrator, Buzzer, LED) is used to provide information to users in multiple ways for people to adapt for different environments; a processing module (8 bit 8051 core microprocessor CC1110F32, 32 K ROM, 4 K RAM, DMA, 12 bit ADC) to perform the sensor data fusion, gesture recognition, and wireless communication control; a RF transceiver integrated in the same microprocessor CC1110 working at 433 MHz for communications; a power regulation and charging module for the compact size 3.7 V lithium polymer rechargeable battery.

**Figure 4.**
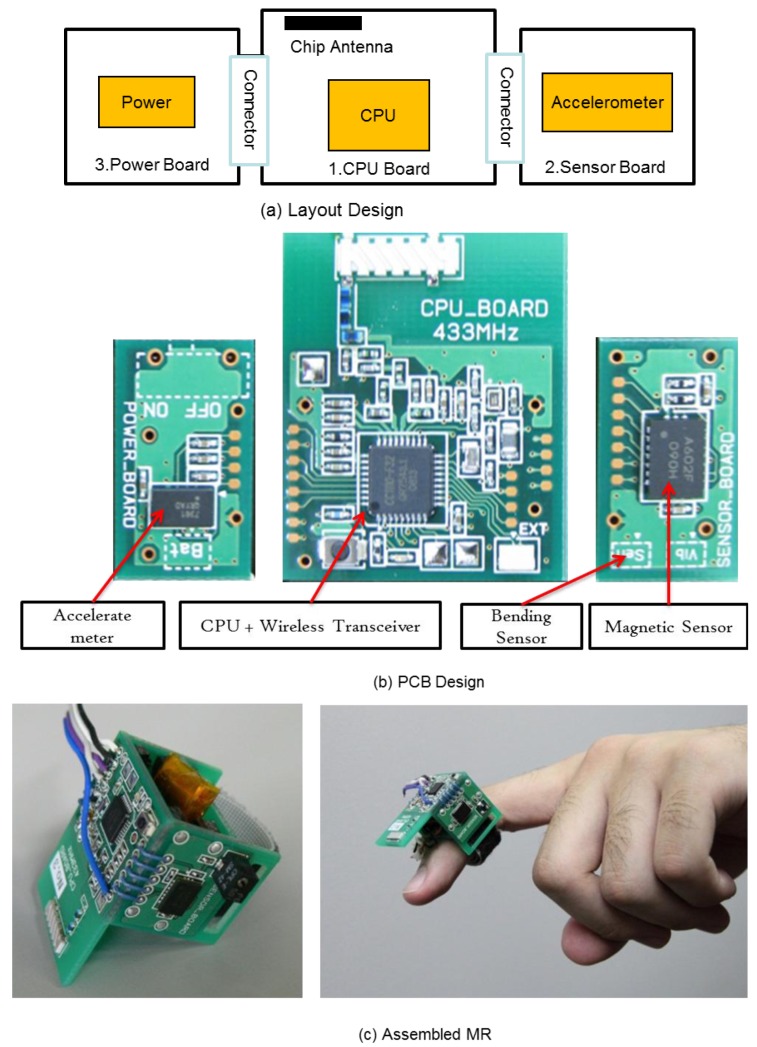
Magic ring: wearable sensing device for finger gesture detection.

To minimize the size of MR, these modules are deployed on three rigid four-layer PCBs (shown in Figure 4(a) Layout Design). The picture of the PCBs is shown in Figure 4(b) PCB Design. Moreover, the PCBs are assembled into a ring shape as shown in Figure 4(c) Assembled MR, so that the relative position and orientation between the finger and accelerometer can be fixed to ensure the correct gesture recognition.

The total weight of the MR (with battery) is about 10 grams, which is on same level as a normal ring on a person's finger. The dimensions are 20 × 25 × 35 mm (height × width × length), which is bigger than a normal ring, but it is still small enough to be worn on the finger without hindering usage of the finger. Moreover, for current development stage, a bigger size is more convenient for the debugging operations. The size of the MR can be further minimized by adopting the ASIC circuit design and increasing the folds or density of the PCB boards.

The minimum wireless communication distance of MR is more than 10 meters in the typical indoor environment like a home, office, or hospital. The maximum distance is dependent on the surrounding environment.

### 4.3. Hardware Structure of EA-Node

The EA-Node different from normal electrical appliances in the sense that it has a Receiver, to bridge the target electric appliance and MR. As shown in the bottom part of Figure 3, the Receiver consists of three major modules: (1) wireless transceiver; (2) processing module to decode and execute the according command; (3) Input/Output (IO) control module customized for connecting and controlling of the target electrical appliance(s). In our experiments, a wireless embedded platform called CuteBox is adopted as the Receiver. Detailed information on CuteBox can be found in [24].

## 5. Static Gestures

### 5.1. Definition of Gesture Vocabularies

**Figure 5.**
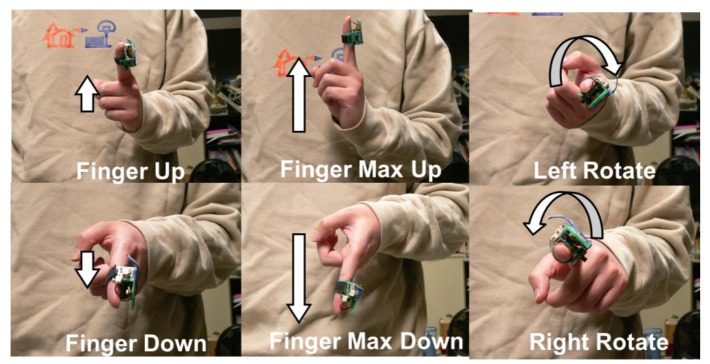
Name and trajectory of the six gestures.

Three pairs of static gestures including Finger Up (FU), Finger Down (FD), finger Max Up (MU), finger Max Down (MD), and Left Rotate (LR), and Right Rotate (RR), are defined, which is suitable for the MR. The postures of these gestures are shown in Figure 5.

A gesture represents some meaning or semantic. In the experimental system, the same gesture can be interpreted as different meanings for different appliances. A semantic mapping table between gesture vocabularies and control commands for each appliance, which is adopted in the evaluation experiments, is given in Table 1.

### 5.2. Gesture Recognition Method

An accelerometer is used to detect the predefined static gestures. The acceleration range of the accelerometer is set to ±6 g (gravity unit: 1 g = 9.8 m/s^2^). The sampling rate of sensor data is 100 Hz (100 times per second). A short time standstill at a position is used to annotate the end of a gesture. If the pitch and rotate degree are within a given range of a predefined gesture, then the gesture is identified and the according command will be automatically triggered.

In details, when the sensor is static, the acceleration sensor on the MR can measure the tilt angle with reference to the ground plane of the Earth. The vector of gravity consists of three components on the three axes of the acceleration sensor. The three components can be used to estimate the degree of pitch and rotate according to formula (1):

(1) $$\begin{array}{l} \alpha =\sin^{-1}(AY/g)\\ \beta =\sin^{-1}(AX/g\times \cos\alpha ) \end{array}$$

where AX, AY is the acceleration on the x and y axis, g is the gravity, *α* and *β* represent pitch and degree of rotation, respectively.

Therefore, the six static gestures can be recognized in a straightforward way. Recognition of dynamic finger gestures will not be discussed in this paper. Early research on the use of the MR to recognize continuous dynamic gestures can be found in [25].

## 6. Target and Function Selection

MR and EA-Node provide a fundamental hardware interface for the universal appliance control method. Furthermore, the next problem is how to abstract the various processes of appliance control and define a unified set of control commands using the MR and EA-Node. In this research, the control process is abstracted into two phases: target selection (TGTSELT) and function selection (FUNSLET). TGTSELT focuses on the selection of one target from surrounding multiple target appliances; FUNSLET focuses on selection of one function from multiple functions on one appliance.

### 6.1. Target Selection

Regarding the selection of control target, there can be three kinds of possible methods: a direct pointing method based on a directional electromagnetic wave such as an infrared beam; a direct triggering method based on the one-to-one mapping between command and control target like hotkeys; a switching method based on a predefined turn-taking protocol. Among the three options, the pointing method requires the target in Line-of-Sight (LoS), which is contrary to the eyes-free features of MR operation. The direct triggering method is the most efficient, but it increases the burden for people to remember more commands. Thus, it is suitable for a situation with just a few targets, or a combination with a switching method to provide quick selection of the most frequently used appliance(s). In our experiment, the switching method is adopted since the pointing method and direct triggering method have been widely explored by other researchers.

The target selection process is illustrated in Table 1. Two gestures, Max Up and Max down, are used for target switching. Each gesture is uniquely identified in the system by a Gesture ID.

Polling protocol is adopted to take turns. The MR is designated as the master node, which polls each of the EA-Nodes in a round-robin fashion. When MR is powered up or reset, the default target of control is null (Target ID 0×00). Once the target switching command is performed, MR will give the next turn to the neighbor node according to target list.

**Figure 6.**
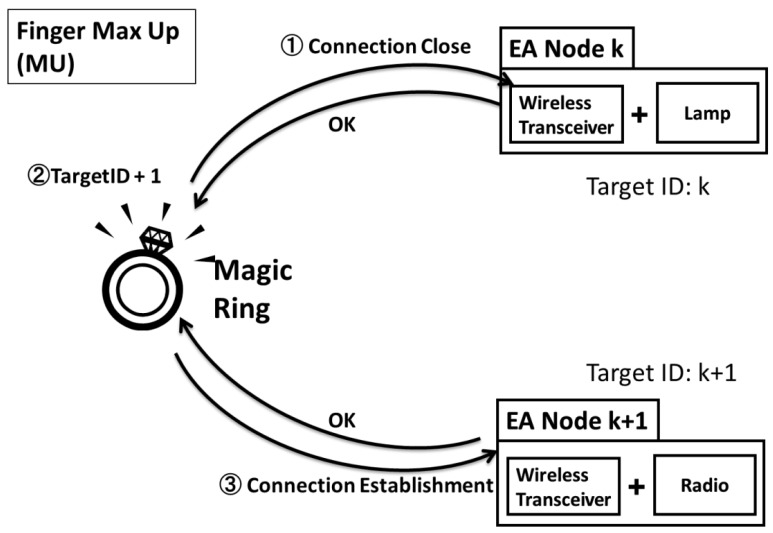
An example of target switching process: finger Max Up (MU) is performed for the switching of the control target from EA-Node k to EA-Node k + 1.

In detail, the target switching process consists of three steps as shown in Figure 6: close current connection, select next appliance, and establish the new connection. For example, the current control target is a lamp which Target ID is k. When gesture “Max Up” is performed, the Gesture ID of MU (e.g., 0×05) is sent to the lamp first to close the current connection. Then MR selects the next Target ID (k + 1) on the target list to establish the new connection.

### 6.2. Function Selection

Generally, an appliance can provide multiple functions like power on/off, volume up/down, switching channels, *etc*. After the selection of a control target, the specific function can be selected among the functions set using protocol of FUNSELT.

Regarding the function selection, there are two possible ways: Full Function Selection (FFS) and Major Function Selection (MFS). FFS means the organization of all the functions of an appliance into some structure so that all of them can be selected. MFS means provide the ways of major function selection like a hotkey.

#### Full Function Selection

An appliance can be represented as a set of functions, and these functions form a tree structure according to the functional dependency relationship. Each node of tree is a function, which can be controlled by a pair of contrary command like on/off, faster/slower, bigger/smaller, *etc*. The root node F1 is power on/off.

Typically, five commands are enough for the function navigation and selection: two commands for vertical navigation between multiple layers, two for horizontal navigation between functions in the same layer, and one for selection.

FFS provides the ways to access all of the functions, but it has two disadvantages. First, it shows low efficiency as the depth and breadth of tree increments. Second, its operation is not intuitive, especially when the traditional display-based interface is not always available.

#### Major Function Selection

From daily experience, the access frequency among different functions is extremely different, such as for a TV, channel selection and volume adjustment are used every day, but white balance adjustment (a function in each TV for color correction) is seldom touched. Thus, for different appliances, major functions can be selected according to the access frequency and meet most of the user's needs. In the remainder of paper, the MFS is adopted to evaluate the usability of MR-based gestural interface.

## 7. Results and Discussion

### 7.1. Evaluation Metrics

Usability of the proposed control paradigm is mainly evaluated through operational efficiency and learning curve. These features were evaluated through a comparative study between the MR and RC. The specific definition of these features is as follows:

Operational efficiency: the average number of tasks that can be completed in a fixed period of time using a controller.

Learning curve: change of the average learning rate in a series of practice sessions using a same controller.

Basically, operational efficiency and learning curve can identify the speed of control and learnability of using such a device, respectively.

### 7.2. Experiment System Setup

**Figure 7.**
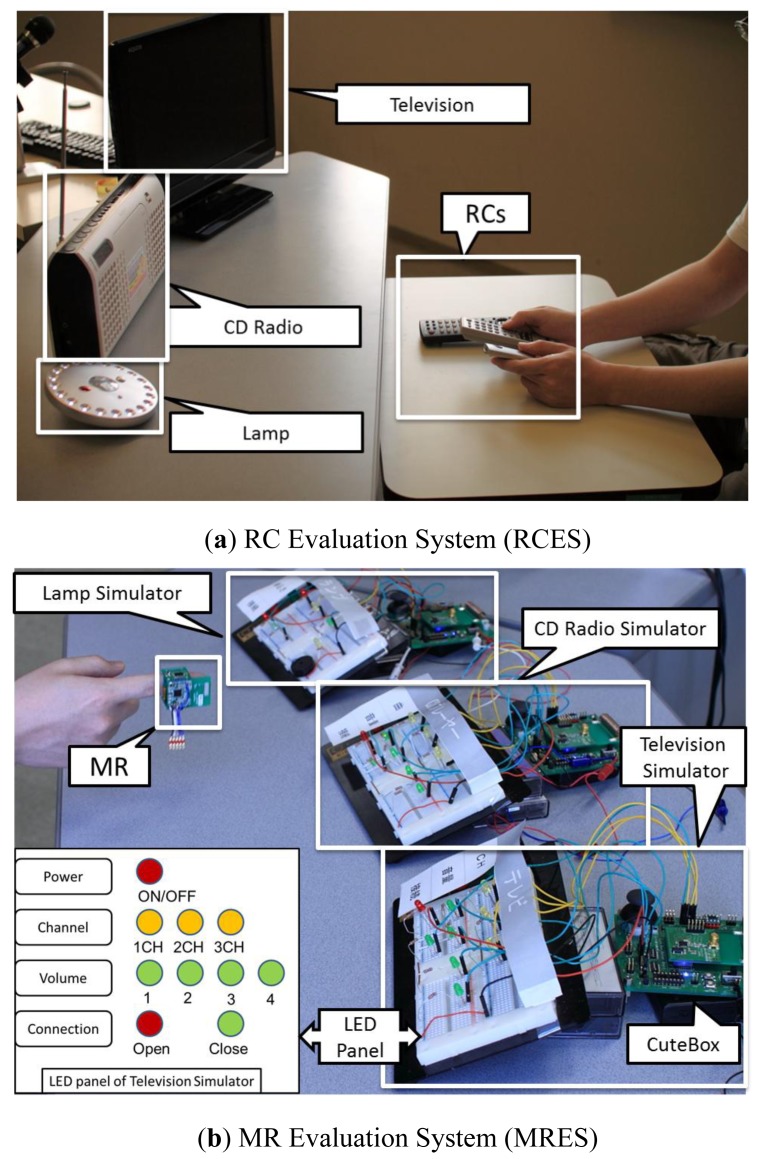
Setting of the two comparative evaluation systems.

Two experimental systems were set up in the research laboratory to evaluate the performance of the MR and RC, respectively, as shown in Figure 7.

#### Remote Control Evaluation System (RCES)

RCES consists of three home appliances including a television, a CD radio, and a lamp, as shown in Figure 7(a). These appliances are set in front of the participant during the experiment. The RCs of these appliances are put at-hand of the participants.

#### Magic Ring Evaluation System (MRES)

MRES consists of three Command Actuator (CA) connected appliance simulators and one MR. A simulator is a dummy appliance, which consists of a CuteBox and a LED panel as shown in the bottom left of Figure 7. CA is implemented on CuteBox, which is a wireless embedded platform for agile development [24]. LED panel is used to feedback the internal state of the simulator to the user. The three states of LED (On, Off, and Flashing) indicate the selected, unselected, and current function, respectively.

### 7.3. Participant

In total, eighteen participants (six female, 12 male, age: 25 ± 4) took part in the evaluation experiments. All participants had no prior knowledge of the MR based gestural interface.

### 7.4. Experiment Process and Setting

#### Process

The basic process consists of 10 sessions (2 minutes per session), and a short time break after each session. The first half part of the sessions (sessions RC1st to RC5th) and the second half of the sessions (session MR1st to MR5th) were assigned to use the RC and MR, respectively.

Only one participant at a time was arranged to perform the experiment under the guide of an observer. At the start of an experiment, the observer will take one or two minutes to briefly explain the usage of the experimental systems. Then, during each section, the participant performs the tasks given by the observer one-by-one. The log data including the task ID and amount of accomplished tasks are recorded by the observer, which are mainly used to evaluate the operational efficiency and learning curve.

#### Task difficulty control

The number of tasks accomplished in a fixed period of time is closely correlated with the difficulty of the tasks. The difficulty in every section should be controlled to ensure an objective experiment result. A random drawing method is adopted for difficulty control of tasks in each session.

First, the tasks are predefined and printed on the cards (one task per card). Totally, the same set of tasks is used for both RC and MR based experiments. The list of tasks is given in Table 2 for reference.

Second, the cards are grouped according to the difficulty level. The tasks are divided into three difficulty levels according to the steps for a given task as in Table 2. The low level only needs one action step; middle level needs two steps; a high difficulty level needs three or four steps to complete a task.

Third, each section is divided into three sub-sessions (40 seconds per sub-session), and the observer use stopwatches for time control. For the 1st, 2nd, 3rd sub-session, the observer randomly draws the cards from the low, middle, and high difficulty level, respectively.

### 7.5. Placement of Controllers

Placement of controllers is another factor to measure a participant's performance. In the evaluation, the three RCs were put at hand of the participant as shown in Figure 7(a). During the experiment, participant can put them all in his/her hands. The MR was mounted on the middle phalanx (between the 1st and 2nd joint) of the index finger, consistently in the evaluation MR sessions, as shown in Figure 7(b).

### 7.6. Experiment Results

The average number of completed tasks and standard deviation (sd) are listed in Table 3. The average performance during the five sessions (10 minutes) across all participants is 9.3 tasks per session, which is about 56% compared with the RC based control.

Three plus factors for RC and two negative factors for MR can be found from the experimental setting. First, participants are all familiar with the RC-based control paradigm. Once they become familiar with the arrangement of the buttons on the RCs, almost all of them maintained a stable high operational efficiency (average tasks 16.5, and sd is 2.5, which is about 15.2% of the average performance as shown in Table 3). Second, the command recognition accuracy is nearly 100% for RC, while it is about 85% for the current MR as mentioned in [25]. Third, the access time for the RC is not included. All the RCs are hand-held in the experiments, but in daily life, RCs are placed somewhere in a room; users spend much more time on finding the RC before starting the control operation.

Meanwhile, two negative factors exist for the MR. First, the MR-based gestural interface is a new control paradigm, and participants need more time to become familiar with it. From the 1st to 5th session, the average ratio of MR to RC increased from 50% to 64%, which indicated that MR shows a competitive performance once users become familiar with such a paradigm through practice. Second, the recognition accuracy of tilt-based gesture detection is error prone since it is tended to be affected by the unstable posture of participants. That is why the average SD of the MR is 3.6, which is about 38.7% of its average performance and 23.7% higher than that of the RC.

## 8. Conclusions

A gesture based method for multiple appliance control is proposed and evaluated. Compared with an infrared remote controller, a competitive performance can be achieved after several minutes of practice. There is some scope for improvement of the operational efficiency and learnability. Our future work will focus on providing personalized gesture vocabularies and improving the recognition accuracy. The usage of the finger-based gestural interface is not only confined to remote control, but also can be applied to broader application fields like under-water communication, the military, robotics, elder care, *etc*.

## Figures and Tables

**Table 1.** Semantic matching between gestures and control commands.

**ID**	**Name**	**Home appliance**		
		**Lamp**	**CD Radio**	**TV**
0×01	Finger Up (FU)		CD selection(+)	Channel(+)
0×02	Finger Down (FD)		CD selection (−)	Channel(−)
0×03	Right Rotate (RR)	Brightness up	Volume up	Volume up
0×04	Left Rotate (LR)	Brightness down	Volume down	Volume down
0×05	Max Up (MU)	Appliance list(+)	Appliance list(+)	Appliance list(+)
0×06	Max Down (MD)	Appliance list(−)	Appliance list(−)	Appliance list(−)

**Table 2.** Task list for evaluation experiment.

**Task ID**	**Task contents**	**Difficulty level**

1	Lamp⇒Change Brightness one time	Low
2	Lamp⇒Change Brightness two times	Middle
3	Lamp⇒Change Brightness three times	High
4	TV⇒Power ON/OFF	Low
5	TV⇒Change Channel one time	Low
6	TV⇒Change Channel two times	Middle
7	TV⇒Change Channel three times	High
8	TV⇒Change Volume one time	Low
9	TV⇒Change Volume two times	Middle
10	TV⇒Change Volume three times	High
11	TV⇒Change Volume four times	High
12	Radio⇒Power ON/OFF	Low
13	Radio⇒Change Channel one time	Low
14	Radio⇒Change Channel two times	Middle
15	Radio⇒Change Channel three times	High
16	Radio⇒Change Volume one time	Low
17	Radio⇒Change Volume two times	Middle
18	Radio⇒Change Volume three times	High
19	Radio⇒Change Volume four times	High

**Table 3.** Tasks completed using MR and RC for all 18 participants.

**Sessions**	**RC (mean ± sd; round off to one decimal)**	**MR (mean ± sd; round off to one decimal)**	**Ratio (MR/RC; %, round off to integer)**

1st	14.3 ± 2.6	7.2 ± 2.8	50%
2nd	16.1 ± 2.2	8.7 ± 3.3	54%
3rd	16.9 ± 2.2	8.7 ± 4.1	51%
4th	17.1 ± 2.0	10.4 ± 3.5	61%
5th	17.9 ± 2.0	11.5 ± 2.8	64%
All	16.5 ± 2.5	9.3 ± 3.6	56%
